# Spin pump and probe in lanthanum strontium manganite/platinum bilayers

**DOI:** 10.1038/s41598-017-06861-1

**Published:** 2017-07-26

**Authors:** G. Y. Luo, J. G. Lin, Wen-Chung Chiang, Ching-Ray Chang

**Affiliations:** 1 Department of Physics, National Taiwan University, Taipei, 10617 Taiwan; 2 Center for Condensed Matter Sciences, National Taiwan University, Taipei, 10617 Taiwan; 3 Department of Optoelectric Physics, Chinese Culture University, Taipei, 11114 Taiwan

## Abstract

Ferromagnetic resonance driven spin pumping (FMR-SP) is a novel method to transfer spin current from the ferromagnetic (FM) layer into the adjacent normal metal (NM) layer in an FM/NM bilayer system. Consequently, the spin current could be probed in NM layer via inverse spin Hall effect (ISHE). In spite of numerous ISHE studies on FM/Pt bilayers, La_0.7_Sr_0.3_MnO_3_(LSMO)/Pt system has been less explored and its relevant information about interface property (characterized by spin mixing conductance) and spin-charge conversion efficiency (characterized by spin Hall angle) is a matter of importance for the possible applications of spintronic devices. In this work, the technique of FMR-SP has been applied on two series of LSMO/Pt bilayers with the thickness of each layer being varied. The thickness dependences of ISHE voltage allow to extract the values of spin mixing conductance and spin Hall angle of LSMO/Pt bilayers, which are (1.8 ± 0.4) × 10^19^ m^−2^ and (1.2 ± 0.1) % respectively. In comparison with other FM/Pt systems, LSMO/Pt has comparable spin current density and spin mixing conductance, regardless its distinct electronic structure from other ferromagnetic metals.

## Introduction

Nowadays the conventional, charge-based electronics has reached a bottleneck for further development of miniaturizing the devices due to the fatal issues such as thermal fluctuation-induced noises and energy loss by Joule heating. The search for alternative technologies to solve these problems leads to the usage of spin, which holds a great promise for the technologies of reducing power consumption. Charge and spin are two intrinsic parameters of an electron. The central concept here is to take the advantage of fast spin transport without the actual transport (or minimal transport) of charge currents. Thus, the integrated mechanism of generation, manipulation, and detection of spin current has become the most sought after research topic and remains as a major challenge in the field of spintronics^1–4^. Among several mechanisms, two physical effects dominate the conversion between spin current and charge current, namely spin Hall effect (SHE)^5–9^, and inverse spin Hall effect (ISHE)^10^.

SHE is driven by the spin-orbit interaction and plays a key role in converting charge currents into spin currents which could not be easily detected^6^. Whereas ISHE converts spin currents into charge currents, offering a way to ease off the difficulty of detecting spin current in most metals^10–12^. In either SHE or ISHE, the efficiency of spin-charge conversion is characterized by spin Hall angle (θ_ISHE_, defined as the ratio of charge current vs. spin current) which is strongly related to the intrinsic electronic structures of materials.

ISHE has been investigated by means of either nonlocal magneto-transport measurement^12^ or spin pumping ferromagnetic resonance (SP-FMR)^10, 13–17^. The magneto-transport method is a direct probe of spin-charge conversion, but with a disadvantage of quantifying spin current due to the charge scattering at interface. The SP-FMR method, on the other hand, is an effective way of determining θ_ISHE_ and spin diffusion length λ. However, due to the details of measurement or analysis, some disagreements still exist on the experimental values of θ_ISHE_ and λ for the same material. For the value of θ_ISHE_ of Platinum (Pt), the techniques of spin-torque-induced ferromagnetic resonance^18^ and modulation of damping^19, 20^ estimate it from 7.6 to 11%; whereas it varies from 0.67 to 8%^13–17, 21–23^ derived from FMR-SP technique with either coplanar waveguide or cavity mode. Even within the same cavity mode^14, 17^ the obtained θ_ISHE_ is 1.3 and 4% with respect to λ_Pt_ = 7.7 nm and 3.7 nm. On the other hand, the experimental results for the spin pumping efficiency from FM layer to Pt, characterized with spin mixing conductance ${g}_{r}^{\uparrow \downarrow }$, have more or less reached a common consensus. A scaling behavior of ISHE voltage vs. FMR procession angle was observed in FM/Pt bilayers^24^, yielding a value of ${g}_{r}^{\uparrow \downarrow }$ = 4 ± 3 in the unit of 10^19^ m^−2^ as seen in Table 1. A significant difference in the value of ${g}_{r}^{\uparrow \downarrow }$ between Py/Pt and Fe_3_O_4_/Pt may be related to the ferrimagnetic nature of Fe_3_O_4_, which requires more experimental evidences to confirm.

**Table 1:** List of parameters for various FM materials.

FM layer	Spin polarization (%)	Resistivity (μΩ cm)	${g}_{{\rm{r}}}^{\uparrow \downarrow }$(10^19^ m^−2^)	Ref.
Ni	43 ± 2	6.9	4 ± 3	24, 45, 51
Co	42 ± 2	6.2		
Fe	45 ± 2	9.6		
Py	37 ± 5	15		
Co_2_MnSi	56	20	4 ± 3	46, 47
Fe_3_O_4_	−(80 ± 5)	5200	0.3 ± 0.2	24, 48, 49
LSMO	100 ± 5%	1000 ± 5%	1.8 ± 0.4	[24, 30, 47, this work]

The spin polarization was obtained with point-contact Andreev reflection measurement (ref. 45) except LSMO and Fe_3_O_4_ that came from spin-resolved photoemission results (refs 30 and 48). The resistivity of single crystal Py (ref. 43), Co_2_MnSi (ref. 46) and LSMO (ref. 50) at room temperature was listed in the third column. The resistivity of Fe_3_O_4_ (ref. 49) was measured for 100-nm thin film. The values of spin mixing conductance in the second last column were mostly obtained with spin pumping technique (refs 48).

Enlighted by our pioneer work on La_0.7_Sr_0.3_MnO_3_(LSMO)/Pt bilayers^25, 26^, it is realized that a systematic SP-FMR study should include all essential physical parameters for the investigated materials as well as a comprehensive analysis on FMR data, such that the origin of disagreement among reports could be self-prevailed. In this work we utilize the technique of cavity SP-FMR to investigate the intrinsic spin current properties on two series of LSMO/Pt bilayers with varying the thickness of LSMO and Pt. The interest of this study is two folds. First, from the material aspect of view the hole-doped perovskite LSMO is a material less studied in the subject of ISHE^25–29^ but with unique characteristics such as high spin polarization, half-metallic band structure^30^ and room temperature colossal magnetoresistance^31^. It is a potential candidate for spin pump because of its low damping constant. Second, from physics point of view, the controlling parameters of spin Hall angle in different FM/NM bilayers have not been well understood yet, thus more investigation is essential for the future applications.

## Results

### Basic characterizations

**Figure 1:**
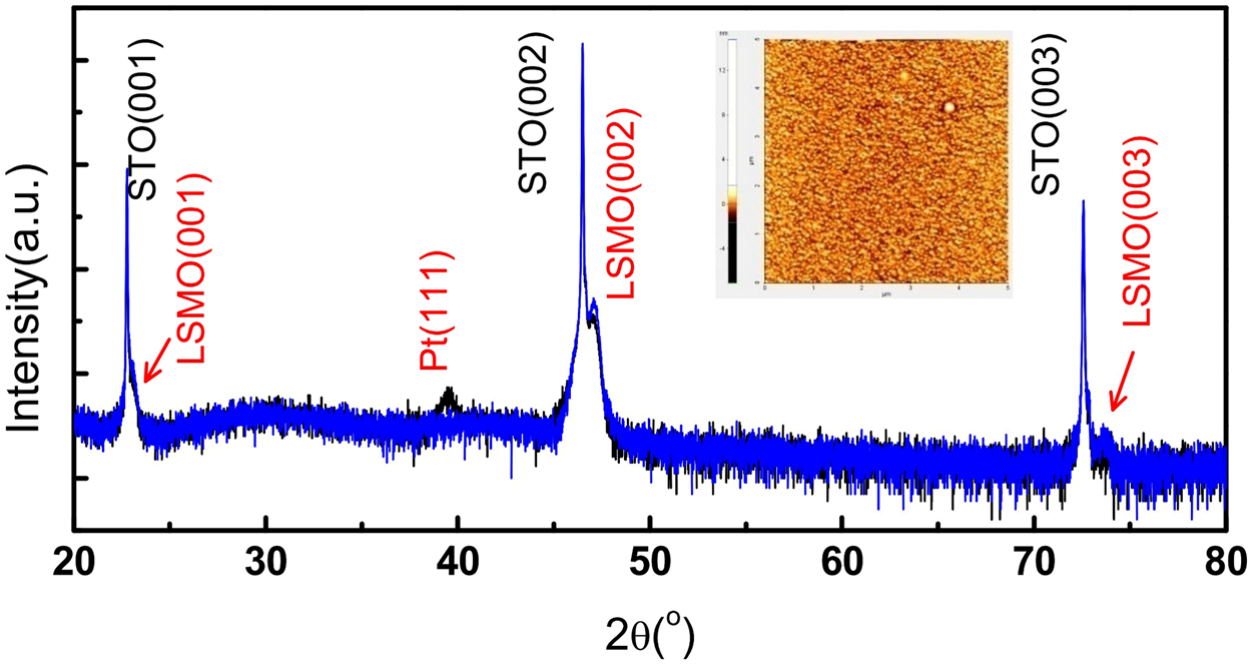
The θ-2θ x-ray diffraction patterns of STO/LSMO(20 nm) and STO/LSMO(20 nm)/Pt(30 nm), represented by the blue and the black curves respectively. The inset shows the AFM image (scanning area 5 × 5 μm^2^) taken on the surface of STO/LSMO(20 nm).

SrTiO_3_(STO) single crystal is chosen for the substrate of all LSMO and LSMO/Pt films. Figure 1 shows the x-ray diffraction patterns for STO/LSMO(20 nm). The blue curve is for STO/LSMO and black one for STO/LSMO(20 nm)/Pt(30 nm). Within the scan range, three sharp peaks corresponding to the (001), (002) and (003) reflections of STO are observed for both samples. The main peaks of LSMO appear next to them and are marked as LSMO(001), LSMO(002) and LSMO(003), indicating that the LSMO layer grows along the STO(001) direction. The two curves are almost identical except that the black curve has an additional peak at 2θ ~ 40°, corresponding to Pt(111). The inset of Fig. 1 displays the AFM (atomic force microscopy) image taken on the surface of STO/LSMO(20 nm). The topological information indicates that the LSMO film is rather smooth with a root-mean-square (rms) roughness around 0.4 nm within the scanning area of 5 × 5 *μ*m^2^.

**Figure 2:**
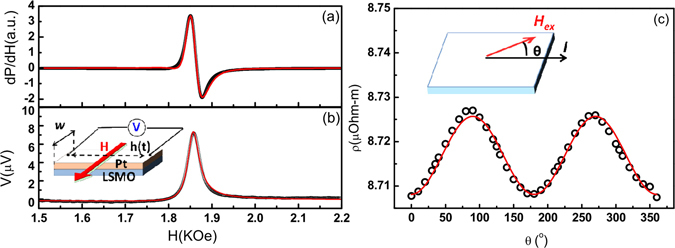
(**a**) The derivative FMR absorption spectrum of LSMO (20 nm)/Pt (6 nm). The red curve indicates the best fit using the first derivative of a Lorentzian function. (**b**) The corespondent voltage spectrum of LSMO (20 nm)/Pt (6 nm). The red curve is the fitting with Eq. 1. The inset illustrates schematically the spin pumping mechanism and the setup of the voltage measurement with the direction of field assigned. *H*_*ex*_, *h*_*m*_(*t*), *t*_*LSMO*_ *(t*_*Pt*_ *)*, and *w* are the external magnetic field, the time dependent microwave magnetic field, the thickness of LSMO(Pt) layer and the width of bilayer respectively. (**c**) The black open circles are the data of angular dependent resistivity of 20-nm LSMO and the red curve is the best fit to cos^2^θ. The inset shows the configuration of measurement with *I* denoting the applied current. θ is the angle between *I* and *H*_*ex*_ on the film plane.

The FMR spectrum and voltage are recorded simultaneously on the same bilayer samples. Figure 2a shows the derivative FMR spectrum as a function of *dc* magnetic field (H) for a typical LSMO(20 nm)/Pt(6 nm) bilayer. The corresponding voltage (V) vs. H is plotted in Fig. 2b. The rise of V at resonant field is a direct response of spin pumping. The asymmetric Lorentzian-like curve of the V- spectrum is decomposed into two parts^13^, i.e. the symmetric FMR-induced ISHE voltage (*V*_*ISHE*_) and the asymmetric term *V*_*AHE*_ from the anomalous Hall effect (AHE), as described by

1 $$V(H)={V}_{ISHE}\,\frac{{\rm{\Delta }}{H}^{2}}{{(H-{H}_{R})}^{2}+{\rm{\Delta }}{H}^{2}}+{V}_{AHE}\,\frac{-2{\rm{\Delta }}H(H-{H}_{R})}{{(H-{H}_{R})}^{2}+{\rm{\Delta }}{H}^{2}}$$

where Δ*H* and *H*_*R*_ are the linewidth and the resonant field respectively. The phase of *V*_*AHE*_ originated from the interaction between the microwave electric oscillation and the magnetization of the FM layer has an intrinsic π/2-difference with *V*_*ISHE*_ at resonant condition, which is the main cause for the asymmetric part of the voltage spectrum.

**Figure 3:**
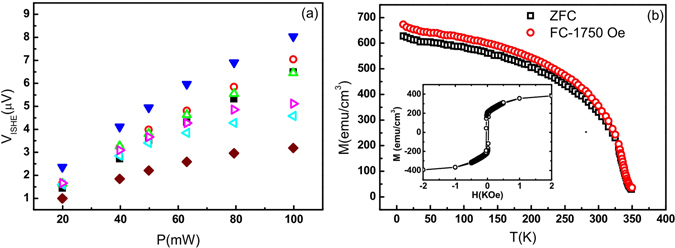
(**a**) Microwave power dependence of *V*_*ISHE*_ for the series of LSMO/Pt(*t*_Pt_) bilayers with different *t*_Pt_: 4 nm (**▪**), 6 nm (**○**), 8 nm (**Δ**), 11 nm (**▾**), 17 nm (**◁**), 21 nm (**▷**) and 30 nm (**◆**). (**b**) Field-cooled and zero-field-cooled magnetization vs. temperature for LSMO(20 nm)/Pt(6 nm). The inset shows the room-temperature hysteresis loop of the bilayer.

The value of *V*_*ISHE*_ is shown in Fig. 3a with the variation of excitation microwave power from 20 mW to 100 mW for the entire series of LSMO/Pt(*t*_Pt_) bilayers. A simplified equation ${j}_{s}^{0}\propto {h}_{m}^{2}(1+{H}_{R}/4\pi {M}_{eff})/{(1+2{H}_{R}/4\pi {M}_{eff})}^{2}$ is adopted, where ${j}_{s}^{o}$ is the spin current density, *h*_*m*_ the microwave magnetic field and *M*_*eff*_ the effective magnetization. Accordingly, ${j}_{s}^{o}$ should increase linearly with the square of *h*_*m*_, which yields a leaner dependence of *V*_*ISHE*_ vs. microwave power. However, for samples with thicker Pt, *V*_*ISHE*_ saturates at high power due to the heating effect from high microwave power^27^. To demonstrate this, Fig. 3b plots the temperature dependent field-cooled (FC) and zero-field-cooled (ZFC) magnetization for LSMO(20 nm)/Pt(6 nm), showing the magnetization at 300 K is very sensitive to temperature change. Hence, the microwave heating would substantially decrease the magnetization as well as ISHE voltage. In this work, we focus on the linear microwave power regime (<60 mW) where the theory applies.

### Damping constant and effective spin mixing conductance of LSMO/Pt

In the FMR formulation, the total free energy *E* of a thin magnetic system is considered to be second-order and can be described by the following equation^32:^

2 $$\begin{array}{rcl}E & = & -{M}_{s}H[\sin \,{\theta }_{H}\,\sin \,{\theta }_{M}\,\cos ({\phi }_{H}-{\phi }_{H})+\,\cos \,{\theta }_{H}\,\cos \,{\theta }_{M}]+2\pi {M}_{s}^{2}\,{\cos }^{2}\,{\theta }_{M}\,-\,{K}_{\perp }\,{\cos }^{2}{\theta }_{M}\end{array}$$

**Figure 4:**
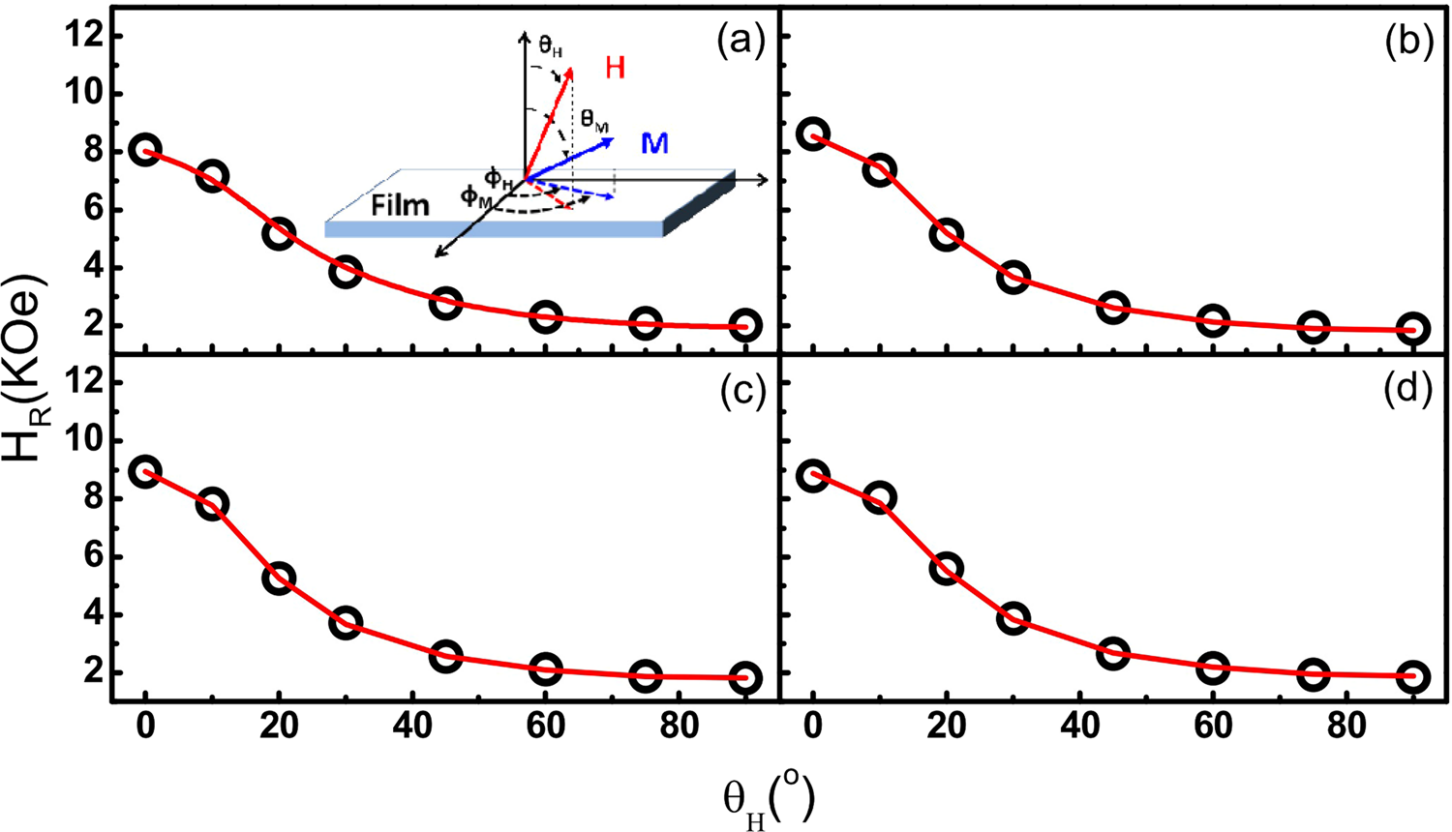
The off-plane angular dependence of *H*_*R*_ for LSMO (*t*_*LSMO*_)/Pt (11 nm) with: (**a**) *t*_*LSMO*_ = 12 nm, (**b**) *t*_*LSMO*_ = 16 nm, (**c**) *t*_*LSMO*_ = 20 nm, and (**d**) *t*_*LSMO*_ = 26 nm. The red curves are the fitting results using Eqs 5 and 6. The inset in (**a**) indicates the coordinate system used for analysing the ferromagnetic resonance.

The coordinate system of the above expression is demonstrated in the inset of Fig. 4 with *H*, *M*_*s*_, and *K*_⊥_ representing the external magnetic field, the saturation magnetization vector, and the perpendicular uniaxial anisotropy constant, respectively. *E* includes the contribution of Zeeman energy, demagnetization energy and perpendicular anisotropy energy. The resonance field *H*_*R*_ is determined by the following resonance condition^33–36^

3 $$(\omega /\gamma )=\frac{1}{(Ms\,\sin \,{\theta }_{M})}{({E}_{{\theta }_{M}{\theta }_{M}}{E}_{{\phi }_{M}{\phi }_{M}}-{E}_{{\theta }_{M}{\phi }_{M}}^{2})}^{1/2}$$

where *E*_*ij*_(*i, j* = *θ*_*M*_ or *φ*_*M*_) denotes the partial derivative index of *E*. The equilibrium state of magnetization is obtained by setting $\partial E/\partial {\theta }_{M}=0$ and $\partial E/\partial {\phi }_{M}=0$, and is expressed as^31^

4 $$2{H}_{R}\,\sin ({\theta }_{M}-{\theta }_{H})=4\pi {M}_{eff}\,\sin (2{\theta }_{M})$$

In Eq. 4, the effective magnetization *M*_*eff*_ is defined by *M*_*eff*_ = *M*_*s*_ − 2 *K*_*⊥*_ */*4*πM*_*s*_. By applying Eq. 3, the resonance condition is deduced

5 $$\begin{array}{rcl}(\omega /{\gamma }^{2}) & = & {H}_{1}\times {H}_{2}\\ \,\,\,\,\,\,\,{H}_{1} & = & ({H}_{R}\,\cos ({\theta }_{H}-{\theta }_{M})-4\pi {M}_{eff}\,{\cos }^{2}\,{\theta }_{M})\\ \,\,\,\,\,\,\,{H}_{2} & = & ({H}_{R}\,\cos ({\theta }_{H}-{\theta }_{M})-4\pi {M}_{eff}\,\cos \,2{\theta }_{M})\end{array}$$

**Figure 5:**
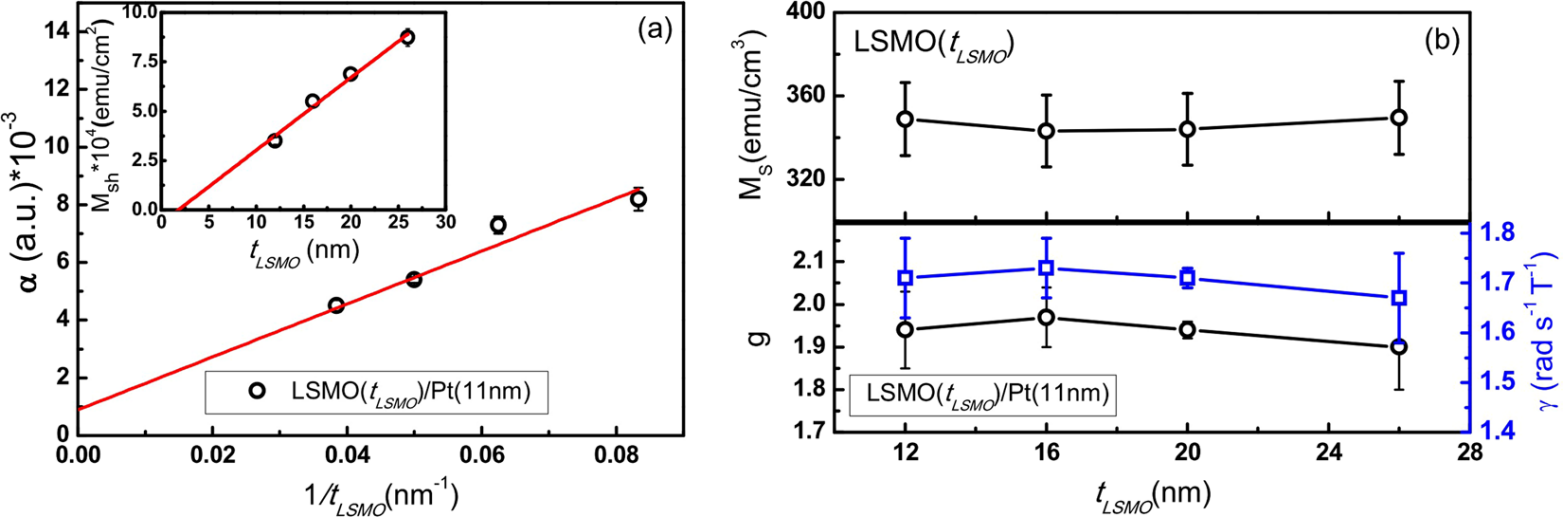
(**a**) The plot of damping constant vs. 1/*t*_*LSMO*_ for LSMO(*t*_*LSMO*_ = 12, 16, 20 and 26 nm)/Pt(11 nm) bilayers. The red line represents the linear fit. The inset of (**a**) shows the thickness dependence of the sheet saturation magnetization (M_Sh_). The intersection of linear fit yields a magnetic dead layer of 1.7 nm. (**b**) The upper panel shows the bulk saturation magnetization M_S_ for LSMO of different thicknesses. The lower panel shows the fitting results for gyromagnetic ratio (γ) and the corresponding g-factor.

In our FMR measurements, the microwave power is set at 40 mW while the out-of-plane field angle with respect to the film plane *θ*_*H*_ is varied from zero to 90 degrees. The angular dependent *H*_*R*_ is plotted in Fig. 4a–d for the four bilayers of different LSMO thicknesses (*t*_*LSMO*_). The red curves in Fig. 4 are the fitting results using Eqs 4 and 5. From the fitting results, the gyromagnetic ratio *γ* is obtained. The damping constant *α* is deduced using *α* = *γ*∆*H*/*ω*, where ∆*H* is the FMR linewidth and *ω* is the microwave frequency. Figure 5a shows that *α* value of bilayer is inversely proportional to *t*_*LSMO*_. The saturation magnetization *M*_*s*_ and the effective *g*-factor (*γ* = *gμ*_*B*_/*ћ*) of four LSMO films are plotted in Fig. 5b as function of *t*_*LSMO*_. Both *M*_*s*_ and *g*-factor are independent of *t*_*LSMO*_, with their mean values being 350(3) emu/cm^3^ and 1.94(2) respectively. The g-factor is slightly smaller than 2 in consistent with previous report^37^, which may originate from the spin-orbital coupling and the partial quench of orbital moment at interface as the case in Ni-Fe film^38^. Based on spin pumping model^39, 40^, the total magnetic relaxation of a bilayer can be described by Gilbert damping

6 $$\alpha ={\alpha }_{0}+\frac{\gamma }{4\pi {M}_{s}}\frac{\hslash }{{t}_{FM}}{g}_{r}^{\uparrow \downarrow }$$

where *α* is the Gilbert damping constant, *α*_0_ is the intrinsic damping constant *ћ* is the reduced Plank constant, *t*_*FM*_ is the thickness of the FM layer. *α*_0_ in the right side of Eq. 6 comes from the intrinsic FM relaxation and is expected to be equal to the value measured in a single FM layer, whereas the second term is due to the interfacial relaxation. Based on the linear fit in the data of *α* vs. 1/*t*_*LSMO*_ as shown in Fig. 5, ${g}_{r}^{\uparrow \downarrow }$ = (2.2 ± 0.4) × 10^19^ m^−2^. If one considers the effect of magnetic dead layer at the STO/LSMO interface^41, 42^, which is estimated to be 1.7 ± 0.2 nm by fitting the *t*_*LSMO*_ dependence of the sheet saturation magnetization M_Sh_ as shown in the inset of Fig. 5a, ${g}_{r}^{\uparrow \downarrow }$ reduces from (2.2 ± 0.4)×10^19^ m^−2^ to (1.8 ± 0.4)×10^19^ m^−2^.

### Spin hall angle and spin diffusion length of Pt

Two other important parameters, *θ*_*ISHE*_ and *λ*_*Pt*_, are extracted from the Pt-thickness dependent *V*_*ISHE*_ in LSMO(20 nm)/Pt(*t*_Pt_). *θ*_*ISHE*_ is defined by

7 $${j}_{c}=\frac{2e}{\hslash }{\theta }_{ISHE}\,{j}_{s}\times \sigma $$

where *j*_*c*_, *j*_*s*_ and *σ* are the charge current density, the spin current density and the spin polarization vector of the spin current, respectively. *j*_*c*_ can be experimentally determined from the charge current (*I*_*c*_) with an equivalent circuit model^15^: *V*_*ISHE*_ = *I*_*c*_ *R*_*FM*_ *R*_*N*_ */(R*_*FM*_ + *R*_*N*_ *)* = *j*_*c*_ *t*_*N*_ *l/(t*_*FM*_ */ρ*_*FM*+_ *t*_*N*_ */ρ*_*N*_ *)*, where *R*_*FM*_ *(ρ*_*FM*_ *)*, *R*_*N*_ *(ρ*_*N*_ *)*, *t*_*N*_ and *l* are the resistance(resistivity) of the FM layer, the resistance(resistivity) of the NM layer, the NM thickness and the bilayer length, respectively. Accordingly, the value of *j*_*c*_ depends on the resistivity of LSMO and Pt. The resistivity of LSMO(20 nm) is 1.3 ± 5% mΩcm which is very close to the bulk value of 1.1 ± 5% mΩcm. Whereas for Pt films, 36.6 ± 5% μΩ-cm is the average value which is three times of the bulk value (10.8 μΩcm)^43^. The high resistivity is likely related to the polycrystalline nature of Pt films. It is worthy to note that the ρ_Pt_ is roughly a constant value of 32.5 ± 5% μΩ-cm but start increasing in the film thinner than 11 nm. *ρ*_*Pt*_ is 37.8 ± 5% μΩcm for Pt(8 nm), 43.9 ± 5% μΩcm for Pt(6 nm) and 44.6 ± 5% μΩcm for Pt(4 nm), implying a high surface roughness for ultra-thin films.

On the other hand, the value of *j*_*s*_ depends on modeling. According to spin pumping model the spin current density at interface ${j}_{s}^{o}$ (z = 0) could be explicively calculated using FMR technique. It is determined by the dynamics of magnetization and ${g}_{r}^{\uparrow \downarrow }$ with an expression:${j}_{s}^{0}=\frac{\omega }{2\pi }{\int }_{0}^{\frac{2\pi }{\omega }}\frac{\hslash }{4\pi }{g}_{r}^{\uparrow \downarrow }\frac{1}{{M}_{s}^{2}}{[\mathop{M}\limits^{\rightharpoonup }(t)\times \frac{d\mathop{M}\limits^{\rightharpoonup }(t)}{dt}]}_{z}dt$. The expression in an intuitive form is derived by including several physical parameters^13, 14^

8 $${j}_{s}^{o}=\frac{{g}_{r}^{\uparrow \downarrow }{\gamma }^{2}{h}_{m}^{2}\hslash [4\pi {M}_{eff}\gamma +\sqrt{{(4\pi {M}_{eff}\gamma )}^{2}+4{\omega }^{2}}]}{8\pi {\alpha }^{2}[{(4\pi {M}_{eff}\gamma )}^{2}+4{\omega }^{2}]}$$

**Figure 6:**
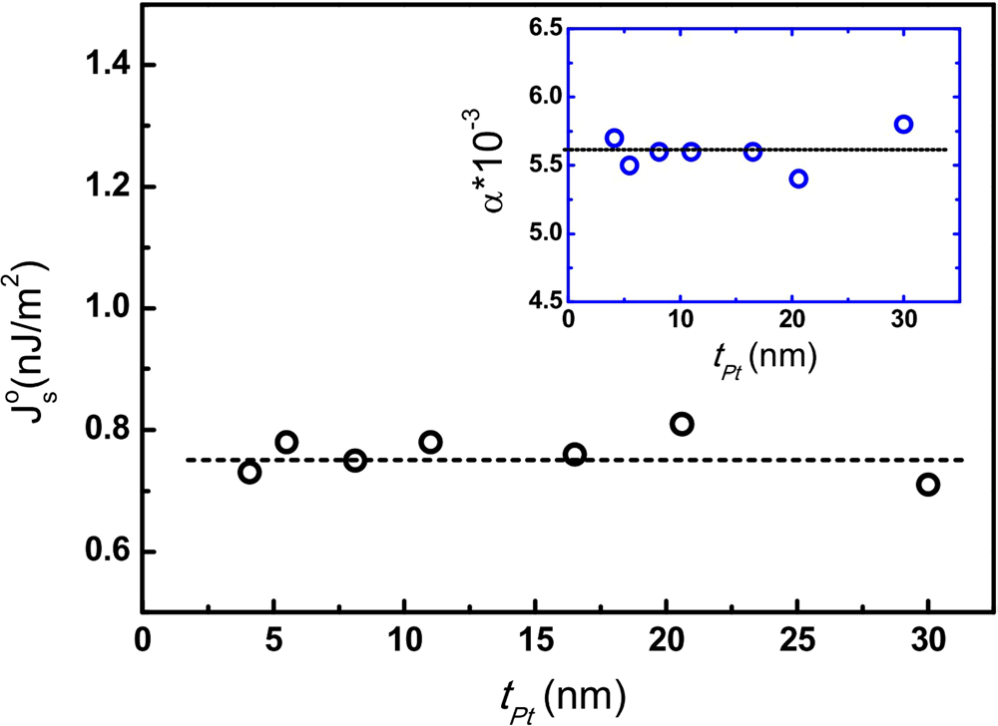
Spin current density ${j}_{s}^{0}$ for the series of LSMO(20 nm)/Pt(*t*_Pt_) bilayers with *t*_Pt_ = 4 to 30 nm with the respective damping constants being shown in the inset.

The data of *t*_*Pt*_ dependent ${j}_{s}^{o}$ are plotted in Fig. 6 for the series of LSMO/Pt(*t*_*Pt*_) bilayers, indicating that ${j}_{s}^{o}$ is insensitive to the Pt thickness, in consistent with the assumption of spin accumulation model^39^. The estimated spin current density of LSMO/Pt is 0.75 ± 0.05 nJ/m^2^ at 40 mW, comparable to that of Py/Pt (~3.0 nJ/m^2^ at 200 mW)^14^, indicating that LSMO is a potential candidate for high efficiency spin pumping.

However, the spin current density could decay along the perpendicular *z* direction of interface and can be written as^14^

9 $${j}_{s}(z)={j}_{s}^{o}\frac{\sinh \,({t}_{N}-z/{\lambda }_{N})}{\sinh \,({t}_{N}/{\lambda }_{N})}$$

Thus, the average charge current density <*J*_*c*_ *>* is obtained by combining Eqs 7 and 9:

10 $$\langle {J}_{c}\rangle {t}_{N}={\theta }_{ISHE}\frac{2e}{\hslash }{\lambda }_{N}\,\tanh \,(\frac{{t}_{N}}{2{\lambda }_{N}}){j}_{s}^{o}$$

**Figure 7:**
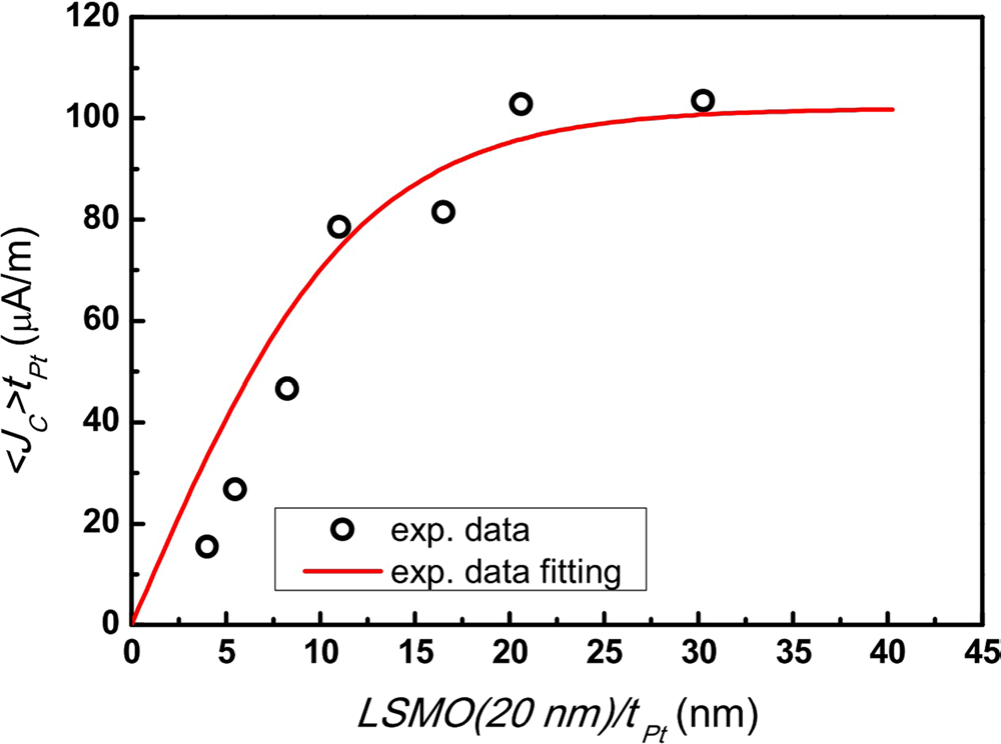
The plot of 〈*j*_*c*_〉*t*_*Pt*_ as a function of Pt thickness. The red curve is the result of a fit with Eq. 10, with an assumption that the spin diffusion length remains constant despite the enhanced resistivity of Pt with a thickness smaller than 11 nm. The fitting yields a spin diffusion length of Pt to be 5.9 ± 0.5 nm.

Eq. 10 represents a simple model that the accumulated spins at the interface diffuse into NM layer and it includes a part of spin current diffusing back into the FM layer. In order to extract the spin diffusion length of Pt (λ_Pt_), the obtained <*J*_*c*_ *>* (by dividing *V*_*ISHE*_ with the total resistance and the width of sample) is fitted using Eq. 10 under the condition of zero-off setting at y axis. The results of fitting are shown in Fig. 7 with the solid line being the fitting result. The fitting is reasonably good except for the thickness thinner than 11 nm. The deviation between data and fitting for thin Pt layer may be due to the interface roughness between Pt and LSMO.

## Discussion

One important issue relating to the validation of ISHE voltage is whether the spin rectification effect (SRE) originating from the anisotropic magnetoresistance (AMR that contains both symmetric and asymmetric components) contribute to the obtained ISHE voltage or not. In our voltage measurements, the contribution of SRE effect could be neglected since the applied magnetic field direction is fixed in-plane, along the short side of the bilayer. Although AMR can be effectively reduced by the experimental setup, the elliptical trajectory of the precession magnetization during the resonance process still leads to AMR contribution, which can be quantitatively characterized as the anisotropic magnetoresistance (*R*_*A*_)^17^. We have obtained *R*_*A*_ = −(0.2 ± 0.1) % for 20-nm LSMO, which is in consistent with the finding for epitaxial La_0.7_Ca_0.3_MnO_3_ film on STO^44^ but one order of magnitude smaller than that of Py (*R*_*A*_~2%) and with an opposite sign^32^. Thus, the AMR effect is neglected in our analysis on ISHE of LSMO.

Table 1 ^24, 45–51^ lists the values of polarization, resistivity and mixing conductance for various materials including LSMO. It shows that the value of *g* ^↑↓^ for LSMO/Pt system agrees with those of most FM/Pt bilayer except that for FM = Fe_3_O_4_, indicating the high values of polarization and resistivity (low conductivity) of LSMO does not affect the value of *g* ^↑↓^ significantly. The values of *λ*, *θ*_*ISHE*_ and *g* ^↑↓^ from different groups are listed in Table 2, showing a significant deviation from various groups which may come from different measurement methods or/and interface properties. As we have clearly demonstrated that the values of *λ*_*Pt*_ and *θ*_*ISHE*_ are correlated since they are from the same fitting curve, it is more reasonable to examine these two values simultaneously. In a SP-FMR study on Co/Pt and Co/Cu/Pt^51^, a universal relation of *λ*_*Pt*_ and *θ*_*ISHE*_ for Pt was found as *λ*_*Pt*_ × *θ*_*ISHE*_ = 0.19 nm. However, a much smaller of *λ*_*Pt*_ × *θ*_*ISHE*_ (0.07 ± 0.01 nm) is obtained from this work based on the values given in Table 2 with *λ*_*Pt*_ = 5.9 ± 0.5 nm. Due to the fact that the interface of LSMO/Pt could be less transparent than other metal/metal interfaces, it is likely that the interfacial spin-memory lost could be the reason^51^.

**Table 2:** Spin diffusion length, spin Hall angle and spin mixing conductance from various research groups are listed.

Material	λ_*Pt*_(nm)	θ_ISHE_ (%)	${{\rm{g}}}_{{\rm{r}}}^{\uparrow \downarrow }$(10^19^ m^−2^)	Method	Ref.
Py/Pt	7.7 ± 0.7	1.3 ± 0.1	3.02	cavity	14
	3.7 ± 0.2	4.0^*^ ± 1.0	2.4	cavity	17
	1.2	8.6 ± 0.5 (2.1 ± 0.5)	3.0	CPW	22, 23
	8.3 ± 0.9	1.2 ± 0.2	2.5 ± 0.2	CPW	21
Co/Pt	3.4 ± 0.4	5.6 ± 1.0	~8.0	cavity	51
YIG/Pt	1.5	11	0.97	cavity	52
	7.3	10 ± 1	0.69 ± 0.06	cavity	53–56
LSMO/Pt	5.9 ± 0.5	1.2 ± 0.1	1.8 ± 0.4	cavity	this work

*The original value in ref. 17 is 0.08 due to a difference in the spin Hall angle definition resulting into a factor of 2. (coplanar waveguide = CPW).

As stated in the above section III, the value of spin current density of LSMO/Pt (0.75 ± 0.05 nJ/m^2^) at 40 mW) is comparable to that of Py/Pt (~3.0 nJ/m^2^ at 200 mW)^14^ which make LSMO a potential material for spin generation. However, this comparison is based on the normalization of ${j}_{s}^{0}\,\,$to microwave power. To be more precise, the effective spin current should be normalized to microwave magnetic field *h*_*m*_ instead of power since the conversion coefficient of *h*_*m*_^2^ to P depend on the type of cavity. In our TE_102_ microwave cavity (Bruker EMX-ER4102ST), the equation is P = 1.4 × *h*_*m*_^*2*^ which converts the power of 40 mW to the *h*_*m*_ value of 0.28 Gauss. For typical TE_110_ as being used in ref. 16, P = 1.5 × *h*_*m*_^2^ and thus the *h*_*m*_ value corresponding to 200 mW is around 0.67 Gauss. However, many ISHE studies did not provide the *h*_*m*_ value such that a precise comparison is not possible.

In conclusion, the spin pump-and-probe experiments are carried out on two series of LSMO/Pt bilayers with the thickness of LSMO and Pt varied. The experimental results are systematical analyzed by the spin pumping model with the consideration of total magnetization relaxation. Three findings from this work are summarized: 1) the comparable mixing conductance of LSMO/Pt with Py/Pt implies that the spin-charge conversion by FMR-SP method is independent of conductivity of FM layer; 2) the fitting result of charge current density vs. Pt thickness in LSMO/Pt yields a spin Hall angle of (1.2 ± 0.1) % which is comparable to that in Py/Pt; and 3) the considerable large spin current density generated in LSMO/Pt reveals that the half-metallic LSMO has potential to serve as a spin pumping source.

## Materials and Methods

One series of single layered LSMO (*t*_LSMO_ = 12, 16, 20 and 26 nm) and two series of bilayers structured as LSMO(20 nm)/Pt(*t*_Pt_ = 4, 6, 8, 11, 17, 21 and 30 nm) and LSMO(*t*_LSMO_ = 12, 16, 20 and 26 nm)/Pt(11 nm) were prepared with a pulsed laser deposition system with a KrF (λ = 248 nm) excimer laser of 160 mJ, at a repetition rate of 1 Hz. The bottom LSMO layer was grown on SrTiO_3_[STO(001)] substrate, which has an in-plane lattice constant of 3.900 Å. The lattice mismatch between STO and LSMO (3.876 Å) is 0.6%, allowing the epitaxial growth of LSMO film at proper deposition conditions. During deposition, the substrate was held at 800 °C in an oxygen environment of 100 mtorr, and subsequently annealed at 400 °C under 760 torr oxygen pressure for 1 hour. The Pt layer was deposited *ex situ* onto STO/LSMO in a commercial sputtering coater (Quorum Technologies Q150TS) having a base pressure of low 10^−5^ torr. The deposition was kept at room temperature with an Ar working pressure of 3.8 × 10^−3^ torr with a deposition rate of 0.22 nm/s, and the lattice constant of Pt layer is 3.924 Å, which is 1.2% larger than LSMO. The crystalline structure of bilayers was confirmed with x-ray diffraction (XRD) on a Bruker-D8 diffractometer using Cu *K*_α_ radiation; whereas the surface morphology was characterized using a Park XEI-100 atomic force microscope (AFM). The saturation magnetization was measured by vibrating sample magnetometer (VSM), and the four-probe method is applied to measure the anisotropic magnetoresistance (AMR) using a Keithley 2182 A nano-voltmeter and a Keithley 2400 source meter.

The bilayer samples were cut into the rectangular shapes of 1.0 × 2.0 mm^2^ to fit in the microwave cavity. To perform ISHE voltage measurements, platinum wires were attached on the Pt-layer surface at the both ends of long side using conducting silver paste. The sample was loaded and positioned at the center of a TE_102_ microwave cavity where the strength of the microwave electric field is a minimum and the magnetic field a maximum. The microwave was provided by an *X*-band Bruker EMX (*f* = 9.8 GHz) system. The DC magnetic field was applied in-plane from 1300 to 2500 Oe, along the short side of sample. ISHE voltage was measured at different microwave power using a Keithley 2182 A nano-voltmeter with respect to sweeping field. All FMR and ISHE measurements were conducted at room temperature.
